# Exercise-Induced Reductive Stress Is a Protective Mechanism against Oxidative Stress in Peripheral Blood Mononuclear Cells

**DOI:** 10.1155/2018/3053704

**Published:** 2018-10-11

**Authors:** Ypatios Spanidis, Aristidis S. Veskoukis, Christina Papanikolaou, Dimitrios Stagos, Alexandros Priftis, Chariklia K. Deli, Athanasios Z. Jamurtas, Demetrios Kouretas

**Affiliations:** ^1^Laboratory of Animal Physiology, Department of Biochemistry and Biotechnology, University of Thessaly, 41500 Viopolis, Larissa, Greece; ^2^Laboratory of Exercise Biochemistry, Exercise Physiology and Sports Nutrition (SmArT Lab), Department of Physical Education and Sport Science, University of Thessaly, 42100 Trikala, Greece

## Abstract

Eccentric exercise is a well-studied modality that induces oxidative stress and muscle damage. Furthermore, it promotes inflammatory response in which peripheral blood mononuclear cells (PBMCs) are the major mediators. Although free radicals are necessary in a specific range of concentrations, yet unknown, it remains unclear whether reductive redox status (i.e., increased antioxidant defenses and impaired free radical generation) is beneficial or not. Thus, the aim of the present investigation was to examine the effects of reductive stress and the impact of reduced glutathione (GSH) baseline values on the ability of PBMCs to counteract oxidative stress induced by a potent oxidative agent. PBMCs were isolated from the blood of subjects who performed eccentric exercise and treated with *t-*BOOH for 24 h. The subjects were clustered in the reductive and the oxidative group on the basis of increased or decreased GSH concentration postexercise compared to preexercise values, respectively. According to our results in PBMCs, lipid peroxidation levels as depicted by thiobarbituric acid reactive substances (TBARS) remained unchanged in the reductive group contrary to the observed enhancement in the oxidative group. In addition, GSH concentration and catalase activity increased in the reductive group, whereas they were not affected in the oxidative group. In conclusion, the effects of an oxidizing agent on the redox status of PBMCs isolated from the blood of athletes after acute eccentric exercise are dependent on the baseline values of GSH in erythrocytes. Otherwise, reductive stress defined by increased GSH levels is a protective mechanism, at least when followed by an oxidative stimulus.

## 1. Introduction

Exercise has been widely associated with free radical generation and oxidative stress induction in an intensity-dependent manner due to diverse mechanisms [1, 2]. Eccentric exercise is a well-studied type of demanding exercise that alters tissue redox status [3, 4]. It is characterized by active contractions and lengthening of the skeletal muscle being, therefore, a severe tissue-damaging exercise modality followed by decreased muscle force production and, finally, inflammation [5, 6]. It is established that muscle damage postexercise initiates a rapid and sequential migration of inflammatory cells from the circulation into the muscle fibers that typically remain there for days [7]. Moreover, reactive oxygen species (ROS) and cytokines produced by the aforementioned cells, when present in specific concentrations, can act as signaling molecules to mediate muscle repair process after a damaging exercise [8]. As a consequence, inflammation or even low levels of oxidative stress are considered beneficial in terms of exercise recovery and tissue repair [9].

During the last decades, there was a common belief that the action of redox status-altering stimuli, such as eccentric exercise and antioxidant supplementation, is preferably assessed in the participants without taking into account the redox individuality [4, 10–12]. However, new, scarce experimental evidence suggests that this might not be the case [3, 4, 13, 14]. These well-designed studies have approached a seemingly paradoxical phenomenon that the differential responses of individuals to such treatments depend on their initial/baseline redox values. Specifically, Block et al. have reported that vitamin C and E supplementation reduces biomarkers of oxidative stress only when their initial values are high [13]. Margaritelis et al. have observed that eccentric exercise induces smaller percent increases in oxidative stress biomarkers (i.e., F2-isoprostanes and protein carbonyls) when their initial values are high and vice versa, whereas glutathione is highly decreased when its initial value is also elevated and vice versa [4]. On the other hand, according to Stagos et al., eccentric exercise induces oxidative stress only in the participants with low baseline antioxidant reserves [3]. Finally, Veskoukis et al. have reported that individuals with low baseline (i.e., resting) reduced glutathione (GSH) levels are linked with decreased physical performance, increased oxidative stress, and impaired redox metabolism of erythrocytes [14]. Intriguingly, administration of N-acetylcysteine (NAC) improved redox status only in the individuals with low resting GSH values and not in those with moderate or high resting GSH values providing novel integrative evidence and formulating a new regime regarding antioxidant supplementation [14]. Thus, it is apparent that baseline values of redox biomarkers should be taken into account in order to state whether a redox stimulus induces either oxidative or reductive stress.

Exercise-induced reductive stress is a rather neglected biological outcome that has gained many adherents recently [14]. Indeed, experimental evidence generated from our research group supports this hypothesis [3, 12]. The consensus in the field of redox biology until a decade ago was that oxidative stress and free radicals are damaging for normal tissue function. Nevertheless, we know today that they are a necessary premise for fundamental biological procedures, namely, signal transduction [11]. This is the reason for the proposal of a modern, acceptable definition of oxidative stress by Jones in 2006 as “a disruption in redox signaling and control” [15] which replaced the most influential definition of oxidative stress as “a disturbance in the prooxidant-antioxidant balance in favor of the former” given by Sies in 1985 [16]. On the grounds of this idea, it could probably be deduced that reductive stress seems to be better than oxidative stress since there is a lower free radical concentration available; thus, there is no need for the activation of the antioxidant mechanism. But this seems to be far from reality. On the contrary, there are no convincing findings towards either direction. On the one hand, there is compelling evidence that reductive stress may be harmful for eukaryotic cells with specific reference to GSH and oxidized (GSSG) glutathione ratio (GSH/GSSG) [17]. Specifically, during reductive stress and similarly to oxidative stress, the production of ROS overwhelms the ability of the antioxidant mechanisms to scavenge them. Two antioxidant enzymes, glutathione and thioredoxin reductases, donate electrons to O_2_ generating H_2_O_2_ mostly because of the impaired availability of the natural electron acceptors, that is, GSSG and oxidized thioredoxin. Therefore, the high levels of GSH seem to be noxious triggering mitochondrial oxidation and cytotoxicity. In the same line, a recent study which examined this phenomenon has also claimed that low exercise-induced oxidative stress blunts adaptations in antioxidant status [18]. On the other hand, it has been demonstrated that reductive stress postexercise induces beneficial effects [3, 12, 19]. According to the above findings, GSH is situated at the spotlight as it is considered critical for cell redox status since it is an integral oxidant scavenger and is considered as a major regulator of ROS production by mitochondria and subsequently of the control of cellular redox environment [20]. Collectively, all the aforementioned observations reinforce the “redox-optimized ROS balance” hypothesis proposed by Aon et al. and Cortassa et al. according to which both oxidative stress and reductive stress are extreme biological conditions, since antioxidant defenses of cell populations such as peripheral blood mononuclear cells (PBMCs) are probably overwhelmed [21, 22].

It is widely known that PBMCs are blood cells being in the front line of the human immune system [23]. They are the most essential mediators of stress and inflammation by producing cytokines, chemokines, and growth factors that may lead to beneficial or even pathological effects on tissues. Intense bouts of heavy exercise, like eccentric, may lead to a robust increase in circulating PBMCs [24, 25]. Thus, they are susceptible to agents able to alter redox status and can be considered a proper model for testing the biological impact of reductive stress and redox individuality.

Considering the aspect that a normal concentration of free radicals or reactive species in general is desired or even required for normal tissue function and muscle regeneration (which is desired after damaging exercise), it remains unclear whether a reductive redox status is beneficial or not. Thus, the aim of the present study was to examine the effects of reductive stress as well as the impact of redox individuality on the ability of PBMCs to cope with oxidative stress induced by a widely used prooxidant agent [3, 4, 12, 19]. We have approached reductive stress on terms of high GSH (i.e., the most important nonenzymatic antioxidant) levels postexercise compared to preexercise values (the individuals with this trait will hereafter be considered as the reductive group), whereas the group that hereafter will be considered as the oxidative group includes subjects with low GSH levels postexercise compared to preexercise values.

## 2. Materials and Methods

### 2.1. Subjects

Seven male and three female volunteers (age 23.6 ± 0.81 years; age range 21–28 years; height 179.1 ± 1.86 cm; weight 76.86 ± 3.71 kg) participated in the present study. Written consent was provided by all athletes after they were informed about the benefits and risks of the investigation. All subjects confirmed the absence of any history of musculoskeletal injury to their lower limbs. Additionally, smoking, alcohol consumption, and any nutritional supplementation as well as any kind of exercise were prohibited over the last days before and until the experiment was completed. Body mass of the subjects being lightly dressed and barefoot was measured to the nearest 0.5 kg (BeamBalance 710, Seca, United Kingdom). Standing height was also measured to the nearest 0.5 cm (Stadiometer 208, Seca). The procedures were in accordance to the Helsinki declaration of 1964 as revised in 2000 and approval was received by the Human Subjects Committee of the University of Thessaly.

### 2.2. Study Design

**Figure 1:**
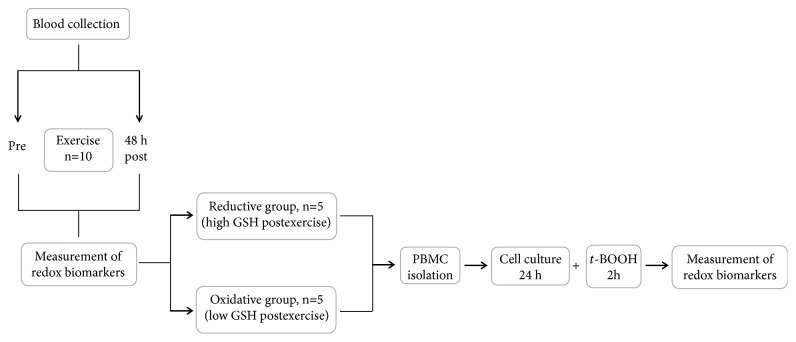
The study design.

All participants performed an eccentric exercise protocol, and blood samples were collected pre- and 48 h postexercise. The subjects were divided in the reductive and the oxidative groups according to the high or low levels of erythrocyte GSH postexercise compared to preexercise values, respectively. Subsequently, PBMCs were isolated from both groups cultivated, at 37°C for 24 h, treated with the potent oxidizing agent *tert*-butyl hydroperoxide (*t*-BOOH) for 2 h, and redox biomarkers were evaluated in the cell lysate. The experimental design is illustrated in detail in Figure 1.

### 2.3. Eccentric Exercise Protocol

Eccentric exercise was performed on an isokinetic dynamometer (Cybex Norm, Ronkonkoma, NY), and exercise protocols were undertaken from the seated position (120° hip angle) with the lateral femoral condyle aligned with the axis of rotation of the dynamometer. Participants were coupled to the dynamometer by an ankle cuff, attached proximal to the lateral malleolus and finally stabilized according to the manufacturer's instructions. Participants completed 5 sets of 15 eccentric maximal voluntary contractions (knee range, 0° full extension to 90° flexion) at an angular velocity of 60°/s. A 2 min rest interval was used between sets, and the total workout time was 15 min. Before the exercise session, subjects performed a 10 min warm-up consisting of cycling on a Monark cycle ergometer (Vansbro, Sweden) at 70–80 rpm and 50 W.

### 2.4. Plasma and Red Blood Cell Lysate (RBCL) Isolation

Ten ml of whole blood was drawn from a forearm vein with subjects in seated position and divided in two ethylenediaminetetraacetic acid (EDTA) tubes. The first one (i.e., 6 ml) was used for PBMC isolation and the second one (i.e., 4 ml) for the determination of redox biomarkers in plasma and erythrocyte lysate. For erythrocyte separation, the tubes were centrifuged (1370 *g*, 10 min, 4°C) and the supernatant (i.e., plasma) was collected. Then, the packed erythrocytes were lysed with 1 : 1 (*v*/*v*) distilled water, inverted vigorously, and centrifuged (4020 *g*, 15 min, 4°C), and the supernatant (i.e., RBCL) was collected. The plasma and RBCL samples were then stored at −80°C prior to biochemical analyses.

### 2.5. Isolation and Cultivation of PBMCs

PBMCs were isolated by Ficoll-Histopaque 1077 density gradient centrifugation according to the manufacturer's instruction. More specifically, 10 ml of whole blood was carefully added into 10 ml of Ficoll reagent and the samples were centrifuged (400 *g*, 20 min, 20°C). The centrifugation was performed without a brake and acceleration in order to separate the monocytes without mixing with Ficoll, which is a cytotoxic agent for blood cells. The monocytes (i.e., lymphocytes, mononuclear cells, and thrombocytes) are located at the semiwhite layer between the plasma and the erythrocytes and Ficoll. The cell layer was carefully harvested by syringe and added to 10 ml of RPMI-1640 medium. The cells were rinsed twice with the medium by centrifugation (400 *g*, 20 min, 20°C) at normal brake and acceleration. The cell pellet after washing was resuspended in 20 ml of RPMI-1640 medium enriched with 1% L-glutamine, 1% antibiotic/antifungal solution, and 10% fetal bovine serum (FBS). The cells were cultured for 24 h in an incubator at 37°C prior to performing the experiments.

### 2.6. Treatment of PBMCs with *t*-BOOH

The cells after isolation and their 24-h incubation were measured using a Neubauer-type cytometer (Hausser Scientific, USA). For the experiments, the cells were plated on a Corning 24-well plate (Sigma-Aldrich, St. Louis, USA). About 1 × 10^6^ PBMCs were added to each position in the 24-well plate diluted in LM-L-glutamine-supplemented RPMI-1640 medium, 1% antibiotic/antifungal solution, and 0% FBS. After 24 h of incubation, the oxidative agent *t-*BOOH was added to the cells to induce oxidative stress. The cells were incubated for 2 h in the RPMI-1640 medium in the absence of serum FBS. The samples containing the cells in the medium were used as the negative control, while the samples containing the cells and the oxidizing agent (80 *μ*M of *t-*BOOH) were used as the positive control. The selected concentration of the oxidizing agent (i.e., 80 *μ*M) did not exert cytotoxic action during the 2 h of incubation, yet induced oxidative stress. Then, the cells were harvested from the plate and centrifuged (3000 *g*, 5 min, 4°C), the supernatant was removed, and the cell pellet was collected. Subsequently, it was washed with 1 ml of phosphate buffer (PBS, 0.01 M, pH = 7.4) at room temperature (RT) and centrifuged (3000 *g*, 5 min, 4°C). The cell pellet was resuspended in 1 ml of PBS, and, finally, PBMCs were ruptured after sonication for 1 min with 10 sec interruptions on ice. Finally, total protein concentration of each sample was determined using the Bradford reagent and the samples were stored at −80°C until further analysis.

### 2.7. Protocols for the Measurement of Redox Biomarkers

To evaluate the participant redox status, GSH concentration and catalase (CAT) activity were determined in RBCL and PBMCs, whereas thiobarbituric acid reactive substances (TBARS) and total antioxidant capacity (TAC) were measured in plasma and PBMCs. All the spectrophotometric assays in the plasma and RBCL were performed with slight modifications as previously described by Spanidis et al. [12] and Veskoukis et al. [26], whereas regarding PBMCs, they were carried out as described below. We should note that the protein concentration of each sample required for the measurement of the aforementioned biomarkers is equal to 30 *μ*g/sample.

For GSH in PBMCs, the reaction was performed in 1 ml containing 520 *μ*l of 67 mM sodium phosphate buffer (pH = 8), 150 *μ*l of cytoplasmic suspension, and 330 *μ*l of 1 mM 5,5′-dithiobis (2-nitrobenzoic acid) (DTNB) solution. The samples were mixed and incubated at RT in the dark for 15 min, and the absorbance was monitored at 412 nm. The concentration of GSH was calculated on the basis of the millimolar extinction coefficient of DTNB (13.6 l/mmol/cm) and is expressed as nmol GSH/mg protein.

For the determination of CAT activity in PBMCs, the reaction was performed in 3 ml containing 150 *μ*l of cytoplasmic suspension and 2845 *μ*l of 67 mM potassium phosphate buffer (pH = 7.4). The samples were incubated for 10 min at 37°C. Then, 5 *μ*l of a 30% *w*/*v* H_2_O_2_ solution was added, and the absorbance was immediately monitored at 240 nm for 1.5 min. The activity of CAT was based on the molar extinction coefficient of H_2_O_2_ (40 l/mol/cm) and is expressed as U/mg protein.

For TBARS, 400 *μ*l of cell suspension was mixed with 500 *μ*l of 35% trichloroacetic acid (TCA) solution and 500 *μ*l of Tris-HCl solution (200 mM, pH = 7.4), and the samples were incubated for 10 min at RT. Then, 1 ml of 2 M Na_2_SO_4_ solution with 55 mM of thiobarbituric acid (TBA) were added, and the samples were incubated for 45 min at 95°C in a water bath. Then, the samples were cooled for 5 min on ice followed by the addition of 1 ml of 70% TCA, the samples were centrifuged (15,000 *g*, 3 min), and the absorbance of the supernatant was monitored at 530 nm. TBARS concentration was calculated on the basis of the micromolar extinction coefficient of malondialdehyde (MDA) (0.156 l/*μ*mol/cm) and is expressed as nmol/mg protein.

For TAC, the reaction was performed in 1 ml containing 50 *μ*l of cytoplasmic suspension, 450 *μ*l of 10 mM sodium phosphate buffer (pH = 7.4), and 500 *μ*l of 0.1 mM 2,2-diphenyl-1-picrylhydrazyl (DPPH^·^) radical solution. Samples containing only the radical solution were diluted in the sodium phosphate buffer (pH = 7.4) and were used as the control. The samples were mixed vigorously and incubated for 60 min at RT in the dark. Then, they were centrifuged (20,000 *g*, 3 min, 4°C), and the absorbance was monitored at 517 nm. TAC was expressed as *μ*mol DPPH^·^ reduced to 1,1-diphenyl-2-picryldrazine (DPPH-H) by the antioxidant components of the cytoplasmic suspension per mg of sample protein.

Total protein of the samples was determined using the Bradford assay [27]. Each assay was performed in triplicates. Blood samples were stored in multiple aliquots at −80°C and thawed only once before analysis. All reagents were purchased from Sigma-Aldrich (St. Louis, MO, USA).

### 2.8. Statistical Analysis

The statistical analysis was based on one-way ANOVA followed by Dunnett's test for multiple pairwise comparisons. The statistical significance level was set at *p* < 0.05. For all statistical analyses, SPSS version 20.0 (SPSS Inc., Chicago, IL, USA) was used. Data are presented as mean ± SEM.

## 3. Results

**Figure 2:**
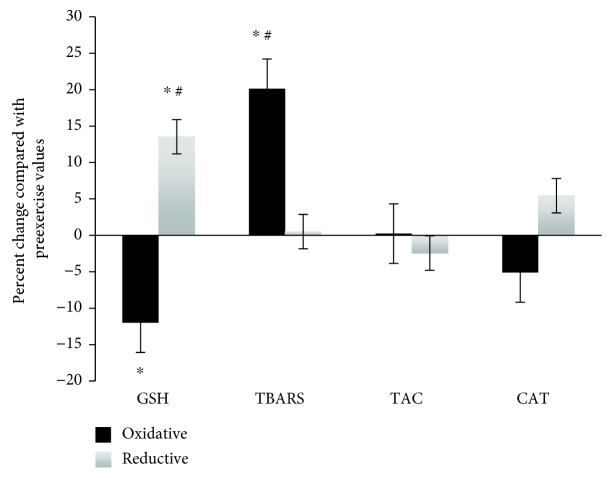
Percent changes of GSH and CAT in RBCL and plasma TBARS and TAC 48 h postexercise compared with preexercise in both oxidative and reductive groups. ^∗^Statistically significant compared with the preexercise value (*p* < 0.05) ^#^Statistically significant difference between the two groups in the same time point (*p* < 0.05). GSH: reduced glutathione; CAT: catalase; RBCL: red blood cell lysate; TBARS: thiobarbituric acid reactive substances; TAC: total antioxidant capacity.

The participants were divided on the basis of those who exerted increased GSH concentration in RBCL postexercise by 13.5% (i.e., the reductive group, *n* = 5) and those with decreased GSH concentration in RBCL postexercise by 12.1 (i.e., the oxidative group, *n* = 5) compared with the preexercise values (Figure 2). These GSH values will hereafter be considered as the initial or baseline values. The athletic history of the participants was not considered as a criterion since the reductive group constituted 3 individuals who performed regular resistance training, while 2 individuals had never been in contact with any kind of exercise. The inverse proportion existed in the oxidative group, which constituted 2 well-trained and 3 nontrained individuals. With respect to our results, an expected significant difference was observed in GSH levels between these two groups (i.e., reductive and oxidative). Similarly, a significant difference between the two groups was also observed in TBARS, as they were significantly increased by 20.1% in the oxidative group and they remained unaffected in the reductive group (Figure 2). Therefore, the participants of the reductive group seem to be protected against exercise-induced lipid peroxidation. Moreover, there were no significant changes in TAC and CAT activity between the two groups.

**Figure 3:**
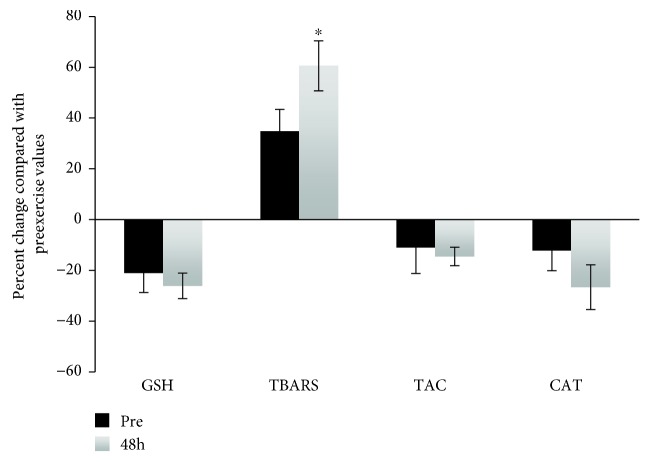
Percent change of the tested redox biomarkers in the *t-*BOOH-treated PBMCs pre- and 48 h postexercise in the oxidative group compared with the control. ^∗^Statistically significant compared with the preexercise value (*p* < 0.05). GSH: reduced glutathione; CAT: catalase; RBCL: red blood cell lysate; TBARS: thiobarbituric acid reactive substances; TAC: total antioxidant capacity.

**Figure 4:**
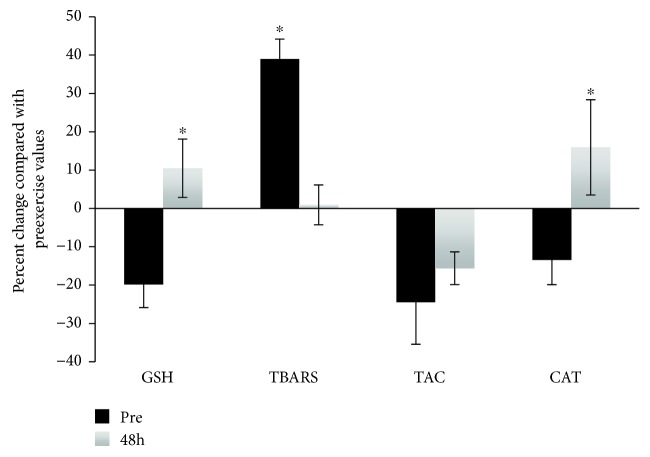
Percent change of the tested redox biomarkers in the *t-*BOOH-treated PBMCs pre- and 48 h postexercise in the reductive group compared with the control. ^∗^Statistically significant compared with the preexercise value (*p* < 0.05). GSH: reduced glutathione; CAT: catalase; RBCL: red blood cell lysate; TBARS: thiobarbituric acid reactive substances; TAC: total antioxidant capacity.

The analyses of the redox biomarkers tested in PBMCs were based on the comparison of their levels in PBMCs treated with *t-*BOOH compared with control cells in both the reductive and oxidative groups. The differences between the two groups were expressed as percent change. With respect to the TBARS levels of the oxidative group, a statistically significant increase was observed 48 h postexercise compared with the preexercise value indicating that eccentric exercise induced a high level of lipid peroxidation (Figure 3). There were no significant changes in GSH, TAC, and CAT activity between the two time points. On the contrary, the analyses in the reductive group revealed statistically significant increases in the GSH and CAT levels 48 h postexercise compared with the preexercise values (Figure 4). In the same pattern, a significant decrease in postexercise TBARS levels was also observed indicating that the lipids in the reductive group are protected against exercise-induced oxidative stress. The latter finding is in contrary with the corresponding data from the oxidative group (Figure 3).

## 4. Discussion

Over the last few years, several studies have made serious efforts to approach “reductive stress,” a condition in which cells exhibit a reductive (i.e., increased antioxidant defenses and impaired free radical generation) redox status after a redox-altering stimulus (e.g., exercise, nutritional, and pharmacological interventions) [14]. In this study, we report that the effects of an oxidizing agent (i.e., *t-*BOOH) on the redox status of PBMCs isolated from the blood of athletes after acute eccentric exercise are dependent on the RBCL baseline values of GSH. As GSH is considered the most important and abundant endogenous nonenzymatic antioxidant [28], we divided the participants in two groups according to their initial (the postexercise as we stated in the results) erythrocyte GSH levels. In particular, half of the participants that have their GSH levels increased 48 h postexercise were clustered in the reductive group. The rest 5 individuals were included in the oxidative group because their GSH concentration postexercise (i.e., GSH baseline values) was decreased. According to our results in PBMCs, lipid peroxidation levels as depicted by TBARS concentration remained unchanged after exercise in the reductive group compared to preexercise, contrary to the observed enhancement in the oxidative group. In addition, the GSH concentration and CAT activity were increased in the reductive group, whereas they were not affected in the oxidative group. It seems, therefore, that reactive species production postexercise followed by administration of an oxidizing agent can be successfully buffered by high baseline levels of GSH sufficiently protecting macromolecules against subsequent oxidative modifications.

It is widely established that eccentric exercise, which was used in the present study in order to group our participants, is linked to remarkable muscle damage [5]. Some common outcomes of eccentric exercise include large elevations of oxidative stress biomarkers and increased levels of creatinine kinase and myoglobin in plasma lasting for days [9]. Furthermore, a previous research of our group has reported that eccentric exercise induces severe delayed-onset muscle soreness accompanied by low antioxidant reserves and impaired reducing activity highlighting the direct connection between muscle damage and the degree of exercise-induced oxidative stress [29]. However, even though eccentric exercise is linked to remarkable muscle damage, recent findings indicate that individuals exhibit significant variations regarding their oxidative status postexercise [4]. Given that eccentric exercise generates greater amount of muscle damage compared to other exercise modalities [9], it becomes apparent that it highly affects exercise-induced oxidative stress and, thus, it is an excellent model for clustering individuals according to high or low values of redox biomarkers (GSH in particular).

The major characteristic of this elaborative study is that we take into consideration the initial values of GSH in order to cluster the participants in the reductive and oxidative groups. This approach has been scarcely adopted by other important papers of the field, as well [3, 4, 13, 14]. A novel feature, though, that this article touches upon is that the GSH baseline values, although referred to postexercise GSH values, they are considered as the initial GSH levels for our subsequent cell culture experiment. According to our findings, this approach is quite reasonable. A paper that has been published recently reported that individuals with low resting (i.e., preexercise) GSH levels are more susceptible to oxidative stress and impaired physical performance compared to their counterparts with high resting GSH values [14]. This finding is in line with the results we present here (i.e., high initial GSH values are linked to protection against oxidative challenge). Although we define initial values differently than Paschalis et al., our results are on the same page. Therefore, we believe that our findings would not be much different if we had grouped our subjects on the basis of resting (i.e., preexercise) values.

It has been reported that strenuous exercise leads to increased circulating levels of proinflammatory mediators, such as PBMCs, and upregulates PBMC growth factor genes like epiregulin and platelet*-*derived growth factor, which are effective in healing wounds and in stimulating muscle cell regeneration [30, 31]. Moreover, it has been stated that PBMCs could also contribute to tissue growth and repair [30]. Given that reactive species are considered crucial signaling molecules for exercise adaptations [32], we wondered whether the reductive group could be benefited from exercise in the same extent compared to their counterparts of the oxidative group. In order to address this issue, we focused on the response of PBMCs isolated from individuals postexercise after their treatment with a potent and widely used oxidizing agent. PBMCs were selected as they are cells of major importance in the human immune system [33]. We hypothesized that PBMCs isolated from reductive volunteers might more effectively counteract the *t-*BOOH-derived oxidation. The data obtained by our experiment confirmed this notion. Intriguingly, in the reductive group, *t-*BOOH led to an induction of CAT and GSH. The induction of both GSH and CAT seems to represent a protective mechanism against lipid peroxidation as the levels of TBARS remained unchanged after exercise even if *t-*BOOH caused severe oxidative response.

Taking into consideration the mechanism of *t*-BOOH action when administered to PBMCs, our results are very interesting. Typically, *t*-BOOH is an organic hydroperoxide that is commonly used as a toxic, redox-altering agent [34]. It is known that *t*-BOOH is metabolized by glutathione peroxidase generating GSSG via GSH oxidation [35, 36]. As it has been previously shown, increased GSH concentration may become harmful for physiological cells by transferring electrons to O_2_ since there are no available natural electron acceptors (e.g., GSSG) as it happens in reductive stress context [17, 37]. Although this could be the case in our study, it seems that the increased baseline values of GSH are not harmful because, due to the presence of a potent oxidizing agent, GSH is oxidized to form GSSG. Thus, reductive stress on the basis of baseline values is perhaps a protective mechanism when followed by a stimulus that induces oxidative stress, such as administration of *t*-BOOH. It has also been proposed that *t*-BOOH interacts with Fe^+2^ leading to the formation of *t*-BO^·^ radicals [36], whereas it can also be converted to peroxyl and alkoxyl radicals by enzymes of cytochrome P450 complex and through free iron-dependent reactions [35]. It is obvious that the production of free radicals can further decrease GSH levels, impair the mechanisms of antioxidant defense, and initiate lipid peroxidation. This decrease in GSH concentration implies that it is a protective mechanism present after the combination of reductive stress with an oxidizing stimulus.

The observed responses could be mainly attributed, at the molecular level, to specific transcription factors, namely, NF-*κ*B, p38 MAPK, members of the FoxO family, and nuclear factor erythroid-derived 2-like 2 (Nrf2) all activated by low ROS levels [38, 39]. Focusing on Nrf2, it has been suggested that it is critical for the regulation of the intracellular redox status [40, 41]. It acts by promoting both constitutive and inducible expressions of the antioxidant response element- (ARE-) regulated genes, which code for numerous antioxidant metabolites and enzymes [42]. Two glutathione-related ARE-regulated enzymes are glutathione reductase (GR) and GSH synthase (GSS) that play significant roles in GSH synthesis and regeneration [28]. Therefore, we can aptly hypothesize that Nrf2 is a key player in the control of exercise and chemically induced shift between oxidative and reductive stress in the examined individuals.

## 5. Conclusion

In conclusion, it is a fact that there is a debate in the pertinent literature whether reductive stress is beneficial or detrimental. In this study, we report that the effects of an oxidizing agent on redox status of PBMCs isolated from the blood of athletes after acute eccentric exercise are dependent on the baseline values of GSH in erythrocytes. Otherwise, on the basis of our results, reductive stress defined by increased GSH levels in erythrocytes postexercise is a protective mechanism, at least when followed by an oxidizing stimulus. These findings can be considered as a starting point in the effort to examine the relatively new concept of postexercise reductive stress and shed light on its potential beneficial impact on blood redox homeostasis using differential experimental treatments.

## Data Availability

All data, tables, and figures in this manuscript are original and are available upon request.
